# Small Object Detection Network Based on Feature Information Enhancement

**DOI:** 10.1155/2022/6394823

**Published:** 2022-06-01

**Authors:** Huilan Luo, Pei Wang, Hongkun Chen, Vladimir Peter Kowelo

**Affiliations:** School of Information Engineering, Jiangxi University of Science and Technology, Jiangxi, China

## Abstract

Due to the small size and weak characteristics of small objects, the performance of existing object detection algorithms for small objects is not ideal. In this paper, we propose a small object detection network based on feature information enhancement to improve the detection effect of small objects. In our method, two key modules, information enhancement module and dense atrous convolution module, are proposed to enhance the expression and discrimination ability of feature information. The detection accuracy of this method on PASCAL VOC, MS COCO, and UCAS-AOD data sets is 81.3%, 34.8%, and 87.0%, respectively. In addition, the detection results of this paper in detecting small objects are slightly (0.2% and 0.1%) higher than the current advanced algorithms (YOLOv4 and DETR, respectively). Moreover, when these two modules are integrated into other algorithms, such as RFBNet, it can also bring considerable improvement.

## 1. Introduction

As one of the basic tasks of computer vision, object detection has been widely used in a variety of scenes, such as object tracking, intelligent monitoring, and automatic driving. With the development of a deep convolution neural network [1, 2], object detection has completed a great leap from the traditional manual feature detection method to the deep learning method of convolution neural network, many object detection algorithms based on deep learning have been proposed. However, most of the existing object detection algorithms [3] can only successfully detect medium-sized objects and large objects in natural scenes, and the detection effect of small objects and dense objects is not satisfactory. The object detection algorithm based on a convolution neural network can be roughly divided into a two-stage detection algorithm [4–6] and a one-stage detection algorithm [7–11]. Among them, the two-stage detection algorithm first generates multiple candidate regions through selective search, then uses a convolution neural network to extract features from the candidate regions, and finally carries out object category estimation and bounding box regression. The two-stage algorithm has high detection accuracy, but the detection speed is slow due to a large number of candidate regions. The one-stage detection algorithm regards object detection as a regression problem, directly extracts the features of the input image, and carries out bounding box regression, which has the advantage of detection speed. These algorithms have achieved good detection results on general data sets such as PASCAL VOC [12] and MS COCO [13], but there are still great challenges in detecting small objects and dense objects. Small objects or dense objects contain a small number of pixels in the original image and carry limited information. After multiple downsampling in the depth network, the resolution is further reduced, resulting in weakening or even loss of feature information and increasing the difficulty of detection. Therefore, small object detection is still a difficult problem to be solved in the task of computer vision.

In recent years, with the continuous development of deep learning, small object detection research has attracted wide attention and has been widely used in urban intelligent transportation, logistics management, agricultural and forestry development, public safety, disaster relief deployment, and other task scenarios. Small object detection research has important research significance and practical application value. Existing small object detection algorithms are mostly proposed on the basis of general object detection methods. They enhance the spatial details and semantic information of small objects through fusing multiscale features [14–16], increasing the receptive field of fine-grained features [17–21], and introducing contextual information around the object [22, 23] to enrich the expression of feature information. In addition, anchor-free algorithms [24], attention module enhancement [25, 26], super-resolution feature representation [27], and data augmentation [28] have also been studied to solve the problem of small object detection.

In order to improve the detection accuracy of small objects and meet the real-time performance of detection, based on the one-stage classical algorithm SSD [7], a small object detection based on feature information enhancement network (FIEN) is proposed in this paper. We have designed the information enhanced module (IEM) and dense atrous convolution module (DAM) in FIEN, which can enhance the context information around small objects and learn the characteristics of the large receptive field, to improve the detection accuracy of small objects. The main contributions of this paper are as follows:

1. Based on the fusion structure of the feature pyramid network [14], we design an information enhancement module. This module adds global and local information branches to enhance the context information of small objects by learning the global information, local information, and multiscale information of input features.
2. In order to reduce the loss of small object information, we propose a dense atrous convolution module, which uses the atrous convolution to obtain the characteristics of receptive fields of different scales and then fuses them. It is worth noting that in this paper, the dense connection is used to obtain the characteristics of receptive fields at different scales, so as to establish a connection for the characteristics of receptive fields at different scales. The receptive field is expanded without adding additional parameters and calculations, so as to improve the detection effect of small objects.
3. The FIEN algorithm proposed in this paper has achieved good detection accuracy on PASCAL VOC [12], UCAS-AOD [29], and MS COCO [13] data sets. At the same time, this paper also integrates IEM and DAM modules into RFB [17] network to further verify their effectiveness.

## 2. Related Works

### 2.1. Small Object Detection Algorithm

According to the definition of SPIE (Society of Photo-Optical Instrumentation Engineers), an object with an object area less than 80 pixels in the image (256 × 256) is a small object. Another method is that according to the definition of the COCO data set, objects with a size less than 32 × 32 pixels are considered small objects.

In recent years, small object detection has attracted extensive attention, but small object detection is very challenging due to its low resolution, less pixel information, and easy to be disturbed by a complex environment. Many scholars have done a lot of research to improve the detection accuracy of small objects. Super-resolution technology is applied to the field of small object detection aiming at improving the resolution of small objects. This technology mainly generated super-resolution feature representation by increasing the resolution of the high-level feature map [27] or by GAN [30, 31], so as to improve the detection results of small objects. Aiming at the problem that small objects carry little pixel information, many studies used the multiscale fusion method to construct features with edge detail information and semantic information, which is conducive to small object detection. At present, there are three methods of feature fusion: element-by-element addition, element-by-element multiplication, and channel splicing. For example, literature [10, 14, 32, 33] added the low-level feature map of different scales extracted by the feature extraction network with the high-level feature map, so as to detect objects of different scales. DSSD [34] added a deconvolution layer on the basis of the SSD [7] algorithm to multiply the high-level features extracted by the deconvolution layer with the low-level features of the same scale, so as to highlight the object area and enhance the detection of small objects. FSSD [16] sampled feature maps of different scales to the same scale for fusion, so as to further enhance the features for detection. In the face of the interference of complex background on small object information, the introduction of an attention mechanism is an effective method. It can make the network pay more attention to the area containing small object information, reduce the impact of background information on detection results, and improve the accuracy of small object detection. Li [35] introduced the channel attention module and used the correlation between channels to selectively enhance the areas rich in discriminant information. YOLOv3-A [36] optimizes the redundant channel problem of different levels of features in the channel attention operation and uses the spatial attention mechanism to obtain the distribution of input features over spatial positions, so as to retain the effective information for the detection process. Besides, the research based on anchor-free [24], data augmentation [28], and other methods has also been used to solve the problem of small object detection. Different from the above methods, FIEN uses two key components: IEM and DAM to obtain more feature information, so as to improve the detection performance of small objects.

### 2.2. Multiscale Information Enhancement

Multiscale information enhancement solves the problem of object scale change by acquiring and fusing the feature information of multiple different scales, so as to improve the final detection results. For example, FPN [14] used the top-down structure with the horizontal connection to detect objects of different scales by using the features of different scales, which effectively solved the problem of scale change. Since then, many research works have improved based on FPN structure, such as adding additional pathways to further fuse deep and shallow features [32, 37]. The attention mechanism [25, 38] is introduced to guide the fusion of feature information at different levels. Moreover, feature maps with different atrous rates are fused to learn multiscale information [17–19]. Methods such as deconvolution and element-by-element multiplication to fuse high- and low-level features [34] are also widely used to enhance multiscale information. Different from the above work, this paper proposes IEM and DAM from the perspective of enhancing the context information around the object and enhancing the receptive field information. On the basis of FPN, the IEM adds two branches: global information branch and local information branch, which enhance the context information of small objects by fusing the global information, local information, and multiscale information around the objects. The DAM uses the atrous convolution with different expansion rates to obtain the features of multiple scales of receptive fields and then splices and fuses them to expand the receptive fields without adding additional parameters and computation.

## 3. Methods

**Figure 1:**
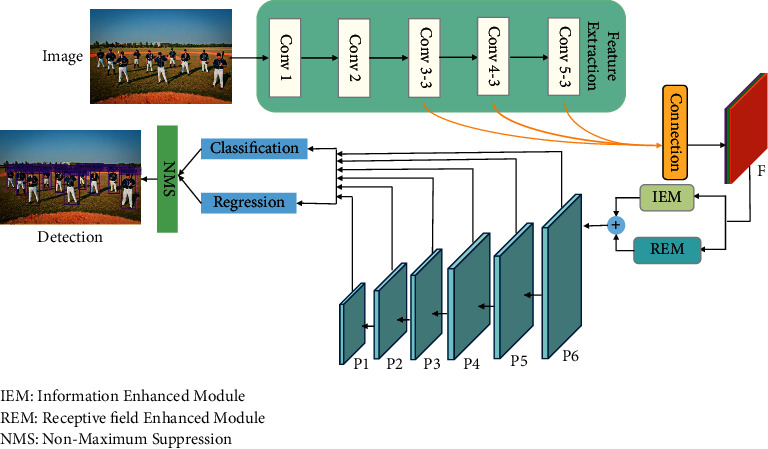
Feature information enhancement network.

SSD [7], as a representative of a one-stage object detection algorithm, has good results in detection accuracy and detection speed. SSD algorithm used large-scale shallow features to detect small objects in the image and large-scale deep features to detect medium-sized or large objects in the image, so as to achieve the purpose of multiscale detection. However, the shallow features of SSD lack the guidance of global semantic information, resulting in the low accuracy of small object detection. In order to improve the ability of SSD to detect small objects, this paper proposes an object detection method based on feature information enhancement on the basis of the SSD algorithm. The network can establish and enhance the exchange and connection between information and produce more discriminative features. The overall structure is shown in Figure 1. Firstly, in order to help locate small objects, we repeatedly use shallow features, up- and downsample both the Conv3-3 and Conv5-3 in the VGG-16 network to the scale of Conv4-3, and then splice the three feature layers in the channel dimension to obtain a multiscale feature map F, which contains texture information and semantic information. On this basis, this paper proposes an information enhancement module (IEM) and dense atrous convolution module (DAM), which use high-level semantic information to guide and enhance the detailed information of shallow small object areas. Based on the enhanced feature map, downsampling is carried out to obtain six feature maps with different scales: *P*6, *P*5, *P*4, *P*3, *P*2. and *P*1, and their sizes are 38 × 38, 19 × 19, 10 × 10, 5 × 5, 3 × 3, and 1 × 1, respectively. The six feature maps with different scales are used for object detection. Multiple priori boxes with different proportions are set at each grid point in each feature map, and multiple boundary boxes are generated through classification and regression. Finally, the boundary boxes obtained from different scale feature maps are filtered out by nonmaximum suppression, and the final detection results are obtained.

### 3.1. Information Enhancement Module

The traditional FPN integrates the semantic information obtained from the high level with the low-level feature map, but the features obtained from the high level only contain the semantic information of a single scale and cannot obtain more comprehensive and richer context information. In order to solve this problem, this paper adopts the structure of feature pyramid attention [38] and designs the information enhancement module, which aims to obtain more semantic information in feature map *F*, fuse feature maps of different scales, and establish semantic communication between information.

**Figure 2:**
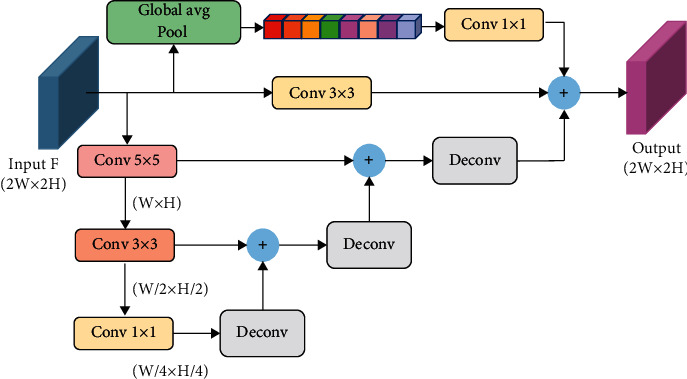
Information enhancement module.

The core idea of the information enhancement module proposed in this paper is to integrate the multiscale semantic information of high-level features and introduce local information and global information at the same time, so as to establish the communication and learning between different information, and this paper uses semantic information to enhance the attention of spatial detail information and generates more discriminative features. This paper assumes that the size of the input high-level feature map *F* is 2*W* × 2*H* × *C*. We obtain global information, local information, and multiscale semantic information through three parallel paths as shown in Figure 2. The calculation process is shown in the following equations:

(1) $$\begin{array}{c} B_{1}=\text{Con}v{\;}_{1\times 1}\left(\text{global}\left(F\right)\right), \end{array}$$

(2) $$\begin{array}{c} B_{2}=\text{Con}v{\;}_{3\times 3}\left(F\right), \end{array}$$

(3) $$\begin{array}{c} B_{3}=\text{FPN}\left(F\right), \end{array}$$

(4) $$\begin{array}{c} F_{o}=\text{Add}\left[B_{1},B_{2},B_{3}\right], \end{array}$$

where *B*_1_, *B*_2_, and *B*_3_ represent the feature map obtained by the first branch, the second branch, and the third branch, respectively; global(∙) represents the global average pooling; FPN(∙) represents the feature pyramid network; Add(∙) represents the addition operation of corresponding elements; and Conv(∙) represents the convolution operation. The first branch adopts global average pooling to obtain the global information of each channel and then adjusts the number of channels through a 1 × 1 convolution layer to fuse and learn the global information of channels. The second branch uses a 3 × 3 convolution to obtain the local information of the feature map. The third branch designs a feature pyramid network, which integrates three different scale features. The feature pyramid network uses a three-level convolution network with a step size of 2, and the size of the convolution kernel is 5 × 5, 3 × 3, and 1 × 1 in turn. The pyramid network fuses the information of different scales in turn, which can more accurately fuse the context information of adjacent scales and get richer multiscale semantic information. Finally, the output features of the three branches are added with the corresponding elements to obtain the final enhanced features. The information enhancement module designed in this paper can integrate different scales of context information and establish the relationship between multiscale information and global and local information, so as to obtain enhanced features with more representation ability.

### 3.2. Dense Atrous Convolution Module

**Figure 3:**
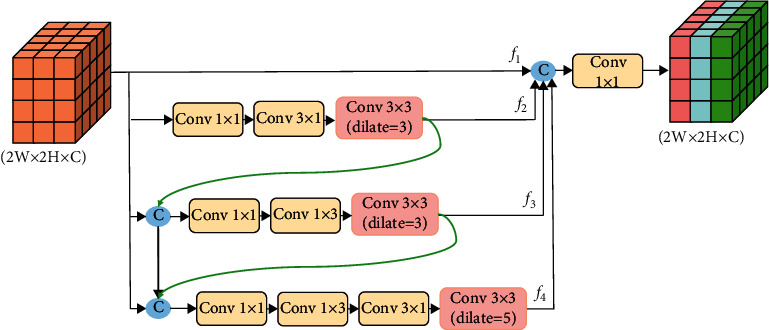
Dense atrous convolution module.

In object detection tasks, there are usually many small objects or objects with large-scale changes. In order to solve this problem, the feature map must be able to cover different scales of receptive fields. Inspired by [39, 40], this paper designs a dense atrous convolution module by using expanded convolution and dense connection, which is used to obtain a denser sampling of high-level features and larger-scale receptive fields, establish and enhance the relationship between different receptive field feature maps, and learn richer information. Its structure is shown in Figure 3, in which four branches are represented as *F*_1_, *F*_2_, *F*_3_, and *F*_4_.

*F*_1_ is the original input feature, which is directly spliced with the output features of the other three branches, so as to further maintain the spatial and semantic information of the original input feature and play the effect of residual connection. *F*_2_ branch aims to enhance spatial information in the vertical direction; firstly, the 1 × 1 convolution is used to reduce the number of channels; then the 3 × 1 convolution is used to perform one-dimensional convolution in the column dimension to enhance the vertical spatial relationship between learning feature points; and finally, the 3 × 3 convolution with the expansion rate of 3 is used to further enhance the context information of learning larger receptive fields. After the output features of *F*_2_ and *F*_1_ are spliced in the channel dimension, enter the *F*_3_ branch. *F*_3_ branch aims to enhance the spatial information in the horizontal direction; firstly, the 1 × 1 convolution is used to reduce the number of channels, and then the 1 × 3 convolution is used to perform one-dimensional convolution in the row dimension to enhance the horizontal spatial relationship between learning feature points, and finally, the 3 × 3 convolution with an expansion rate of 3 is used to further enhance the context information of learning larger receptive fields. *F*_4_ splices the output features of *F*_1_, *F*_2_, and *F*_3_ as input and then convolutes with 1 × 1, 1 × 3, and 3 × 1 convolution, and next 3 × 3 convolution with an expansion rate of 5 is used to enhance the receptive field of the two dimensions of a column vector and row vector of the input feature. Finally, the output features of the four branches are spliced, and then the final output features are obtained by adjusting the number of channels through a 1 × 1 convolution. The calculation process is shown in the following equations:

(5) $$\begin{array}{c} F_{2}={\text{Conv}}_{3\times 3,d=3}\left({\text{Conv}}_{3\times 1}\left(\text{Conv}{\;}_{1\times 1}\left(f_{1}\right)\right)\right), \end{array}$$

(6) $$\begin{array}{c} F_{3}={\text{Conv}}_{3\times 3,d=3}\left({\text{Conv}}_{1\times 3}\left(\text{Conv}{\;}_{1\times 1}\left(C\left\{f_{1},f_{2}\right\}\right)\right)\right), \end{array}$$

(7) $$\begin{array}{c} F_{4}={\text{Conv}}_{3\times 3,d=5}\left({\text{Conv}}_{3\times 1}\left({\text{Conv}}_{1\times 3}\left(\text{Conv}{\;}_{1\times 1}\left(C\left\{f_{1},f_{2},f_{3}\right\}\right)\right)\right)\right), \end{array}$$

(8) $$\begin{array}{c} F_{\text{out}}={\text{Conv}}_{1\times 1}\left(C\left\{f_{1},f_{2},f_{3},f_{4}\right\}\right), \end{array}$$

whereConv_3×3,*d*=3_ and Conv_3×3,*d*=5_ represent the atrous convolution layer, Conv represents the convolution operation, *C*{·} represents the splicing operation along the channel dimension, and *F*_out_ represents the final output feature.

In order to make full use of the specific information learned by each branch and enhance the flow and dissemination of information, the four branches of this module adopt the series mode, that is, the output features of the front branch and the original input features are spliced as the input of the back branch. Reusing the features of the previous branches also avoids the loss of information caused by convolution operation and further enhances the information.

## 4. Experiment and Analysis

The FIEN network proposed in this paper has been tested on PASCAL VOC 2007 [12], MS COCO 2017 [13], and UCAS-AOD [29] data sets. PASCAL VOC 2007 data set has 9,963 images, including 20 categories, of which the number of small objects accounts for about 57%. MS COCO 2017 data set contains 80 categories, 118,287 training images, 5,000 verification images, and 40,670 test images. The images in the data set have complex backgrounds. With a large number of instance objects on each image, the number of small objects is increased, and the evaluation standard is stricter. UCAS-AOD data set is a remote sensing image data set, which only includes planes and cars. However, due to its high-altitude top view shooting, the images have a large field of vision, resulting in many small objects in the images and high background complexity, which brings great challenges to the detection.

### 4.1. Experimental Setup

The experiments in this paper are implemented on PyTorch; the hardware environment is NVIDIA GeForce RTX 2080Ti. In the training process, this paper follows the training strategy of the baseline detector and uses the backbone pretrained on ImageNet, and the loss function is the sum of the location loss function *L*_loc_ and the classification loss function *L*_conf_. The expression is shown in formula (9), where *N* represents the number of a priori boxes. At the same time, the random gradient descent algorithm is used to optimize the weight of the network. The momentum is set to 0.9; the learning rate is set to 0.004; and the decay is set to 0.0005. At the beginning of training, the weight of the model is randomly initialized; if a large learning rate of 0.004 is adopted, the model training may be unstable. In order to ensure the stability of model training, this paper selects the way of warming up the learning rate, as shown in formula (10). Use a small learning rate of 1 × *e*^−6^, and then the learning rate of each epoch increases a little. After six epochs, the learning rate increases to the preset 0.004. At the moment, the preset learning rate is used for training to make the convergence speed of the model faster.

(9) $$\begin{array}{c} \text{Loss}=\frac{1}{N}\left(L_{\text{conf}}+L_{\text{loc}}\right), \end{array}$$

(10) $$\begin{array}{c} L_{\text{warm}}=1\times e^{-6}+\frac{N_{\text{iter}}\times \left(l_{\text{rate}}-1\times e^{-6}\right)}{\text{epoch}\_\text{size}\times 5}, \end{array}$$

where *N*_iter_ represents the number of steps of network training iteration, *l*_rate_ is the initial value of network learning rate, and epoch_size indicates the number of batch sizes contained in an epoch. Considering the limitation of GPU memory, when training the PASCAL VOC data set, set the batch size with the input image size of 300 × 300 and 512 × 512 to 16 and 14, respectively. When training the UCAS-AOD data set, the batch size is set to 16, and when training the MS COCO data set, the batch size is set to 8.

In this paper, the mean average precision (mAP) of various objects is used as the object detection and evaluation index, as shown in the following equation:

(11) $$\begin{array}{c} m\text{AP}=\frac{\sum_{q=1}^{Q}AP\left(q\right)}{Q}, \end{array}$$

where *Q* represents the total number of all detection object categories, *q* represents a certain detection object category, and *AP* represents the average accuracy of a certain detection object category.

The average accuracy *AP* represents the area under the precision-recall curve, and its calculation formula is shown in the following formula:

(12) $$\begin{array}{c} AP=\int_{0}^{1}P\left(R\right)\text{d}R, \end{array}$$

where *P* stands for precision and *R* for recall. The calculation method of precision and recall is shown in the following equations:

(13) $$\begin{array}{c} \text{Precision}=\frac{\text{TP}}{\text{TP}+\text{FP}}\;, \end{array}$$

(14) $$\begin{array}{c} \text{Recall}=\frac{\text{TP}}{\text{TP}+\text{FN}}, \end{array}$$

where TP represents the number of positive samples correctly identified, FP represents the number of negative samples incorrectly identified as positive samples, and FN represents the number of positive samples predicted as negative samples. Positive and negative samples are distinguished according to the selected IOU threshold. Those greater than the IOU threshold are positive samples; otherwise, they are negative samples. In this paper, the IOU threshold is set to 0.5.

### 4.2. Results on PASCAL VOC 2007 Data Set

In order to verify the effectiveness of the FIEN network, this section experiments to train the model on the joint training set of VOC 2007 and VOC 2012 (16,551 images) and test the model on the VOC 2007 test set (4,952 images). During training, the input image size is set to 300 × 300 and 512 × 512, respectively. At the same time, in order to further verify the effectiveness and universality of IEM and DAM, this paper also integrates the two modules into the RFB algorithm for experimental analysis. Due to different experimental environments, SSD [7] and RFB [17] are reproduced in this paper.

Table 1 shows the test results of various algorithms in the VOC 2007 test set. The multiscale test technique is not used in the test of this model. According to Table 1, the results are summarized in the following list:

1. Compared with baseline network SSD and RFB, FIEN and FIEN_RFB have significantly improved detection performance. When the input size is 300 × 300, the overall detection accuracy is improved by 3% and 0.4%, respectively; when the input size is 512 × 512, the overall detection accuracy is improved by 1.5% and 0.4%, respectively.
2. Meanwhile, compared with other algorithms based on SSD networks, such as RSSD [42] and FSSD [16], the performance of FIEN has been shown to be significantly better. At the scale of 300 × 300, mAP increased by 1.7% and 1.4%, respectively, at the scale of 512 × 512, mAP increased by 0.5% and 0.4%, respectively. This shows that the FIEN proposed in this paper can capture more effective information to further improve detection accuracy.
3. As the input scale becomes larger, the detection accuracy of the model is improved. This is because the large-scale image retains more information during network feature extraction, which is conducive to object detection. However, blindly increasing the size of the input image in the process of training and testing will consume more computing resources and time. Therefore, this experiment has only been carried out for 300 × 300 and 512 × 512.

In addition, in order to further explore the detection performance of FIEN for small objects, the detection accuracy of different algorithms in each category is listed in Table 2. The experimental results in Table 2 show that the detection accuracy of the algorithm in this paper is higher than that of the SSD algorithm in all categories, especially in the category of bottle and plant with more small objects. For some categories with a large proportion of small objects, such as boat, chair, and bird, the detection accuracy of FIEN_RFB is 2.6%, 1.4%, and 1.1%, respectively, higher than that of RFB, which shows that the two modules proposed in this paper can extract richer context information, which is conducive to the detection of small objects.

### 4.3. Results on MS COCO 2017 Data Set

MS COCO 2017 is a comprehensive data set including object detection, semantic segmentation, and instance segmentation. For the object detection task, the MS COCO 2017 data set contains a large number of objects with large-scale changes, dense objects, and small objects, including 80 categories, 118,287 training images, 5,000 verification images, and 40,670 test images. The performance evaluation index uses the average accuracy AP and the average recall AR, where IOU = 0.5:0.95 means that 10 thresholds are set in steps of 0.05, and the average value of 10 thresholds is obtained. *S*, *M*, and *L* represent small object, medium object, and large object, respectively.

**Figure 4:**
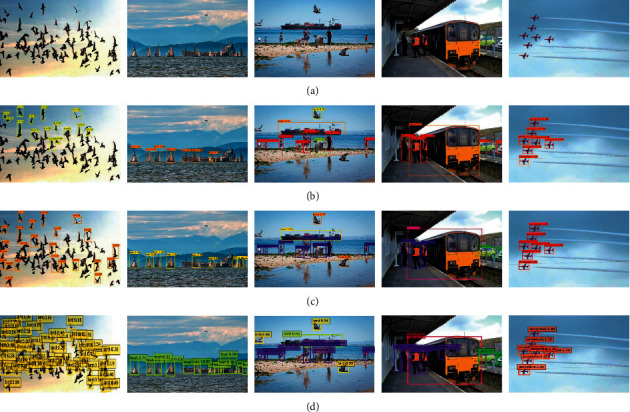
Qualitative test results on MS COCO2017 test dev: (a) image, (b) YOLOv3, (c) YOLOv4, and (d) FIEN512.

**Figure 5:**
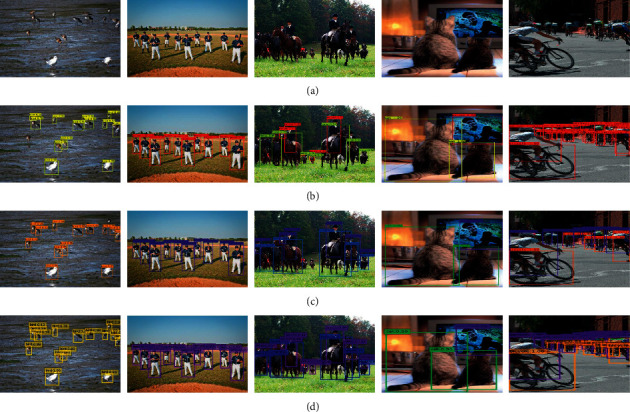
Qualitative test results on MS COCO2017: (a) image, (b) YOLOv3, (c) YOLOv4, and (d) FIEN512.

During the experiment, this paper takes SSD512 as the baseline detector, trains FIEN512, and compares its performance with other algorithms. The experimental results are shown in Table 3; the visual detection results are shown in Figures 4 and 5. According to Table 3 and Figures 4 and 5, the results are summarized in the following list:

1. The detection results of FIEN512 are significantly better than SSD512 [7], YOLOv3 [10], and DSSD513 [34], and the detection results are equivalent to RFB512 [17].
2. Compared with YOLOv4 [11] and DETR [44], the detection result of FIEN512 is slightly lower, but FIEN512 effectively improves the detection accuracy of small objects while ensuring the detection accuracy of large and medium objects, which effectively proves that FIEN network has good advantages in detecting small objects and dense objects.
3. Figure 4 selects images with complex environments, variable object scale, and dense for detection. From the detection results of the fourth and eighth lines, it can be found that FIEN512 has achieved good performance in detecting various types of objects, dense objects, and small objects, which further proves the effectiveness of the FIEN network in detecting small objects and dense objects.

### 4.4. Results on UCAS-AOD Data Set

In order to verify the detection performance of FIEN network for small objects, the UCAS-AOD data set is selected for experiments. UCAS-AOD only contains two types of object and background negative samples of car and plane, with a total of 1,510 images. In this paper, 1,057 images are used as the train set, and 453 images are used as the test set. Although the object type and quantity of the UCAS-AOD data set are far lower than that of the COCO data set, the correlation between objects is strong, which is suitable to verify the effectiveness of this method for small object detection. Although the increase in input scale will improve the detection accuracy, it will also slow down the speed of training and testing. Therefore, this paper only compares FIEN300 with other algorithms in the UCAS-AOD data set. The experimental results are shown in Table 4.

**Figure 6:**
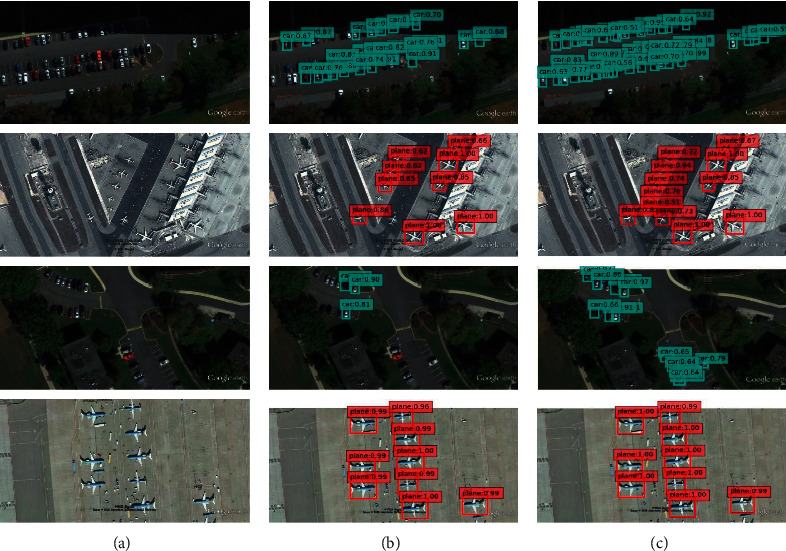
UCAS-AOD test results: (a) image, (b) SSD, and (c) FIEN.

It can be seen from Table 4 that the mAP of the FIEN300 model in this paper on the UCAS-AOD data set is 87.0%, which is 2.8%, 2.3%, and 1.1% higher than SSD300 [7], FSSD300 [16], and MultDet300 [45], respectively, reflecting the superiority of small object detection.  Figure 6 shows some test results of SSD and FIEN on the UCAS-AOD data set. It can be seen from the detection results in Figure 5 that the SSD algorithm has missed detection when detecting small and dense objects. For example, several cars in the third row and the second column are not detected, while using the FIEN method proposed in this paper avoids the occurrence of missed detection, improves the detection accuracy of each object, and can more effectively improve the detection efficiency of small objects.

### 4.5. Ablation Experiment

In order to verify the effectiveness of IEM and DAM in the FIEN network, this section takes the SSD300 algorithm as a baseline detector, integrates the two modules into SSD300, and carries out ablation experiments on the PASCAL VOC 2007 data set.

#### 4.5.1. Verifying the Effectiveness of IEM and DAM

In this experiment, four schemes are used to verify the respective performance of IEM and DAM: (1) baseline SSD300 algorithm is added without any module; (2) only IEM is added to the high-level features of the backbone network; (3) only DAM is added to the high-level features of the backbone network; and (4) add IEM and DAM to the high-level features of the backbone network. The experimental results are shown in Table 5. According to Table 5, the three experimental schemes can improve the detection accuracy of the baseline model, and the detection performance of adding two modules at the same time is the best. This shows that the IEM and DAM modules designed in this paper are effective in capturing context information and establishing the relationship between information. The joint use of the two modules can enhance the performance of the network and improve the detection accuracy of the model.

#### 4.5.2. Verifying the Effectiveness of Each Branch in the IEM

The IEM consists of global information branches, local information branches, and multiscale semantic information branches. In order to further study the detection performance of each branch, this paper designs four types of experiments: (1) only add multiscale semantic information branches; (2) add multiscale semantic information and global information branches; (3) add three branches; and (4) convolution kernels of different sizes are used in multiscale semantic information branches. The experimental results are shown in Table 6. The baseline model is SSD_300 that achieves 77.2% mAP on the VOC 2007 data set. According to the settings in Section 2.1, we first add the multiscale semantic information branch with a convolution kernel size of 3 × 3 to the baseline model, and its performance is improved from 77.2% to 78.4%. Then we use convolution kernels of 5 × 5, 3 × 3, and 1 × 1 instead of convolution kernels of 3 × 3, and the detection performance is improved from 78.4% to 78.6%, which shows that different convolution kernels are used to capture richer information. Then we added the global information branch on this basis, and its detection accuracy reached 79.0%. Finally, after adding local information branches, the detection accuracy reaches 79.3%, which effectively improves the detection performance. In Table 6, C333 and C531, respectively, indicate that the convolution kernel size is 3 × 3 and 5 × 5, 3 × 3, and 1 × 1; glo represents the global information branch; and loc represents the local information branch.

#### 4.5.3. Verifying the Effectiveness of Different Expansion Rates in DAM

In order to explore the influence of different expansion rates of atrous convolution of three branches in DAM on detection performance, this experiment analyzes and compares four different schemes: (1) the expansion rates are 3, 3, and 3 in turn; (2) the expansion rates are 3, 3, and 5 in turn; (3) the expansion rates are 3, 5, and 5; and (4) the expansion rates are 3, 5, and 7. The experimental results are shown in Table 7. It can be found from the results in Table 7 that the best test results are obtained when the expansion rate is set to 3, 3, and 5. The possible reason is that the branches of row spatial relationship and column spatial relationship enhancement learning are suitable for using the same and moderate expansion rate, while the branches of overall spatial relationship enhancement learning need larger receptive fields to obtain richer semantic and spatial information. Therefore, in this paper, the atrous convolution with an expansion rate of 3, 3, and 5 is used to form a DAM in order to better detect objects.

#### 4.5.4. Verifying the Connection Mode of IEM and DAM

**Figure 7:**
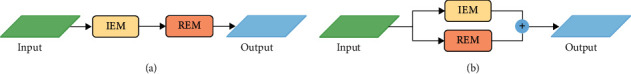
Two connection modes: (a) cascade and (b) parallel.

In order to explore the impact of different connection modes of the two modules on the detection performance, this paper adopts two connection modes for the IEM and the DAM: cascade and parallel, as shown in Figure 7. The final detection results are shown in Table 8. It can be seen from Table 8 that the detection effect of parallel connection for two modules is better than that of cascade connection. This shows that when using cascade connection, due to the small scale of input features, although more abundant information is captured after passing through the information enhancement module, some spatial features of the original features are lost after a series of convolution operations, which cannot provide more useful information for the DAM, resulting in unsatisfactory detection effect. When the parallel connection is adopted, the two modules operate on the input features, and the obtained features do not affect each other. They not only can obtain rich context information but also can establish the relationship between the information. Finally, they are fused to form complementarity and obtain more discriminative features.

## 5. Conclusion

In this paper, we propose a novel small object detection based on a feature information enhancement network (FIEN) with two simple yet effective components to alleviate information loss. Specifically, IEM extends the function of FPN to utilize local and global information in the input feature. Then we introduce DAM to enhance the flow propagation between features and reduce the loss of small object information. Extensive evaluations of three data sets demonstrate that the proposed approach outperforms previous state-of-the-art methods in detecting small objects and the proposed two modules can be well generalized to other algorithms and achieve significant improvement. In addition, the detection algorithm can provide technical support for medical auxiliary diagnosis, intelligent agriculture, automatic driving, and other scenes. Although our method has achieved good results in detecting small objects, there are still some missed detection and false detection in detecting small objects with similar features or occlusion. In future work, we will adjust and optimize the performance of IEM and DAM and verify the generalization of two modules on more detectors. Meanwhile, we will also optimize the composition of the data set and increase the training of occluded small objects.

## Figures and Tables

**Table 1:** Test results on PASCAL VOC 2007 test set.

Method	Backbone	Size	GPU	mAP (%)
YOLOv1 [8]	GoogleNet	448 × 448	Tian X	63.4
YOLOv2 [9]	Darknet-19	352 × 352	Tian X	73.7
SSD300 [7]	VGG-16	300 × 300	2080Ti	77.2
SSD512 [7]	VGG-16	512 × 512	2080Ti	79.8
RefineDet320 [41]	VGG-16	320 × 320	Tian X	80.0
RefineDet512 [41]	VGG-16	512 × 512	Tian X	81.8
RFB300 [17]	VGG-16	300 × 300	2080Ti	80.1
RFB512 [17]	VGG-16	512 × 512	2080Ti	81.2
AFP-SSD [33]	VGG-16	300 × 300	Tian X	79.3
RSSD300 [42]	VGG-16	300 × 300	Tian X	78.5
RSSD512 [42]	VGG-16	512 × 512	Tian X	80.8
FSSD300 [16]	VGG-16	300 × 300	1080Ti	78.8
FSSD512 [16]	VGG-16	512 × 512	1080Ti	80.9
SEFN300 [43]	VGG-16	300 × 300	1080Ti	79.6
SEFN512 [43]	VGG-16	512 × 512	1080Ti	81.2
FIEN300	VGG-16	300 × 300	2080Ti	80.2
FIEN512	VGG-16	512 × 512	2080Ti	81.3
FIEN_RFB300	VGG-16	300 × 300	2080Ti	80.5
FIEN_RFB512	VGG-16	512 × 512	2080Ti	81.6

**Table 2:** Detection accuracy of each category.

Categories	Aero	Bike	Bird	Boat	Bottle	Bus	Car	Cat	Chair	Cow
Small objects ratio (%)	45.8	52.5	59.0	68.9	89.7	43.2	66.1	15.5	78.7	62.9
SSD300 [7]	78.8	85.3	75.7	71.5	49.1	85.7	86.4	87.8	60.6	82.7
RFB300 [17]	83.4	88.0	77.7	74.3	60.5	88.3	87.5	88.1	63.6	86.3
FIEN300	82.3	88.0	80.4	74.4	59.4	87.5	88.1	87.7	65.2	86.6
FIEN_RFB300	84.9	87.7	78.8	76.9	60.7	88.8	87.8	89.2	65.0	86.4

Categories	Table	Dog	Horse	Mbike	Person	Plant	Sheep	Sofa	Train	TV

Small objects' ratio (%)	26.8	22.9	28.7	41.2	58.6	75.8	72.6	17.8	25.7	68.0
SSD300 [7]	76.5	84.9	86.7	84.0	79.2	51.3	77.5	78.7	86.7	76.2
RFB300 [17]	74.6	84.5	88.9	88.2	82.2	55.2	80.3	80.9	88.0	79.8
FIEN300	78.1	86.0	88.3	88.4	81.7	55.5	79.7	80.2	87.6	79.6
FIEN_RFB300	78.1	85.4	89.2	88.9	80.9	55.4	80.6	81.8	88.1	81.0

**Table 3:** Test results on MS COCO 2017 test dev.

Method	Backbone	Size	mAP (%), IOU			mAP (%), area			AR (%), area		
			0.5:0.95	0.5	0.75	S	M	L	S	M	L
YOLOv3 [10]	Darknet-53	608 × 608	33.0	57.9	34.4	18.3	35.4	41.9	—	—	—
YOLOv4 [11]	CSPDarknet-53	608 × 608	41.2	62.8	44.3	20.4	44.4	56.0	—	—	—
SSD512 [7]	VGG-16	512 × 512	28.8	48.5	30.3	10.9	31.8	43.5	16.5	46.6	60.8
RFB512 [17]	VGG-16	512 × 512	34.4	55.7	36.4	17.6	37.0	49.7	27.3	52.3	65.4
RefineDet512 [41]	VGG-16	512 × 512	33.0	54.5	35.5	16.3	36.3	44.3	—	—	—
DSSD513 [34]	ResNet-101	513 × 513	33.2	53.3	35.2	13.0	35.4	51.5	21.8	49.1	66.4
DETR [44]	ResNet-101	—	42.0	62.4	44.2	20.5	45.8	61.1	—	—	—
SEFN512 [43]	VGG-16	512 × 512	33.7	54.7	35.6	19.2	38.0	47.3	29.1	52.5	63.2
FIEN512	VGG-16	512 × 512	34.8	54.8	37.3	20.6	40.0	48.2	30.5	55.0	64.3

**Table 4:** Test results on UCAS-AOD.

Method	Backbone	mAP (%)
SSD300 [7]	VGG-16	81.2
FSSD300 [16]	VGG-16	81.7
MultDet300 [45]	VGG-16	85.9
FIEN300	VGG-16	87.0

**Table 5:** Impact of IEM and DAM on performance.

IEM	DAM	mAP (%)
✕	✕	77.2
✓	✕	79.3
✕	✓	79.6
✓	✓	80.2

**Table 6:** Effectiveness of branches in IEM.

Method	mAP (%)
SSD_300	77.2
SSD_300 + C333	78.4
SSD_300 + C531	78.6
SSD_300 + C531 + glo	79.0
SSD_300 + C531 + glo + loc	79.3

**Table 7:** Effects of different expansion rates on detection performance.

Expansion rate	mAP (%)
3, 3, 3	79.0
3, 3, 5	79.6
3, 5, 5	79.5
3, 5, 7	79.3

**Table 8:** Effects of different connection modes on performance.

Connection modes	mAP (%)
Cascade	79.3
Parallel	80.2

## Data Availability

The data used to support the findings of this study are available at https://host.robots.ox.ac.uk/pascal/VOC/voc2007, https://cocodataset.org/#home, and https://www.ucassdl.cn/resource.asp.
